# Validation of Commercial Activity Trackers in Everyday Life of People with Parkinson’s Disease

**DOI:** 10.3390/s23084156

**Published:** 2023-04-21

**Authors:** Pieter Ginis, Maaike Goris, An De Groef, Astrid Blondeel, Moran Gilat, Heleen Demeyer, Thierry Troosters, Alice Nieuwboer

**Affiliations:** 1 KU Leuven, Department of Rehabilitation Sciences, Neurorehabilitation Research Group (eNRGy), 3000 Leuven, Belgium; 2 KU Leuven, Department of Rehabilitation Sciences, Research Group for Rehabilitation in Internal Disorders (GRID), 3000 Leuven, Belgium; 3 MOVANT Research Group, Department of Rehabilitation Sciences, University of Antwerp, 2000 Antwerp, Belgium; 4 Pulmonary Rehabilitation, Respiratory Department, University Hospitals Gasthuisberg, 3000 Leuven, Belgium; 5 Department of Rehabilitation Sciences, Ghent University, 9000 Ghent, Belgium

**Keywords:** step count, physical activity, physical therapy, wearable sensors

## Abstract

Maintaining physical activity is an important clinical goal for people with Parkinson’s disease (PwPD). We investigated the validity of two commercial activity trackers (ATs) to measure daily step counts. We compared a wrist- and a hip-worn commercial AT against the research-grade Dynaport Movemonitor (DAM) during 14 days of daily use. Criterion validity was assessed in 28 PwPD and 30 healthy controls (HCs) by a 2 × 3 ANOVA and intraclass correlation coefficients (ICC_2,1_). The ability to measure daily step fluctuations compared to the DAM was studied by a 2 × 3 ANOVA and Kendall correlations. We also explored compliance and user-friendliness. Both the ATs and the DAM measured significantly fewer steps/day in PwPD compared to HCs (*p* < 0.01). Step counts derived from the ATs showed good to excellent agreement with the DAM in both groups (ICC_2,1_ > 0.83). Daily fluctuations were detected adequately by the ATs, showing moderate associations with DAM-rankings. While compliance was high overall, 22% of PwPD were disinclined to use the ATs after the study. Overall, we conclude that the ATs had sufficient agreement with the DAM for the purpose of promoting physical activity in mildly affected PwPD. However, further validation is needed before clinical use can be widely recommended.

## 1. Introduction

Maintaining sufficient levels of physical activity (PA) is recognized as an important component in the treatment of Parkinson’s disease (PD) [1,2] and is adopted in current clinical guidelines of physiotherapy [3,4]. A recent retrospective cohort study showed that higher PA levels were associated with better gait and balance scores six years later in PD [5]. Although this class II study should be interpreted cautiously, the case for improving or at least maintaining PA levels in PD is strong [6]. Furthermore, various studies have shown that high PA is associated with a reduced risk for conversion to PD [7,8], and increasing both PA and exercise intensity were recommended as the most important lifestyle changes to delay the onset of PD in prodromal cases [6].

Despite all these positive findings indicating the importance of PA as a strategy to impact disease outcomes it is also known that maintaining or improving PA is not trivial in PD. The same longitudinal study mentioned above [5], demonstrated that PA levels declined significantly over six years in PD, while remaining stable in age-matched healthy controls (HCs). Even in de novo PwPD with low disease severity [9] and in a cohort with mild to moderate disease [10], optimal levels of ambulatory activity were not achieved. Therefore, investigating sustainable strategies to optimize PA levels in PD is paramount.

One possible solution is to provide motivation, feedback, and guidance by monitoring the number of daily steps with wearable devices [11]. Such applications, in conjunction with an expert consulted via remote feedback solutions, have been designed specifically for PD [12] and until now provided modest effects [13]. Another more cost-effective way to tackle the problem is to employ off-the shelf commercial step monitors (i.e., activity trackers—AT) [14,15,16,17] used with or without expert input. An advantage of such an approach is that having an AT is not disease-associated, which helps to counter stigmatization. Importantly, however, given the bradykinetic and sometimes disrupted gait in PD, the validity of these ATs may be inadequate [14]. Yet, this is important for adequately detecting the day-to-day fluctuations in gait quantity and for providing credible feedback to PwPD.

So far, the few validity studies on commercial ATs in PD have included relatively short bouts of continuous walking and this over relatively short distances [14,15,17]. Doing so, Lamont et al. showed that wrist-worn ATs had an error of less than 3% in step counts during self-paced walking in- and outdoors [14]. Wendel et al. [15] and Lai et al. [17] verified these results for both wrist- and hip-worn AT during continuous overground walks of two and six minutes. However, during slower [14] or discontinuous [15] walks, all commercial ATs were less accurate than in situ or video-based counting, suggesting that the quality of the detection may be worse in daily living circumstances [18]. Surprisingly, no study to date investigated whether the daily step count and fluctuations in step count between consecutive monitoring days could be assessed reliably with commercial ATs.

To address this gap, the present study aims to validate two types of commercial ATs in the daily living routine for the first time. The primary objective is to investigate the accuracy and criterion validity of step count detection using commercial wrist-worn (Wrist-AT) and hip-worn (Hip-AT) ATs. We will compare daily step counts obtained from the ATs over 14 days with a those of a research-grade activity monitor (Dynaport Movemonitor; DAM) and contrast the findings between PwPD and age-matched HCs (discriminatory validity). Our hypothesis is that the ATs will underestimate daily steps in PwPD compared to the DAM, while this will not be the case in the HC. Secondary objectives are:

1. To examine whether the ATs can detect day-to-day fluctuations accurately and adequately rank days with high and low step counts when compared with the research-grade device in PD versus the HC.
2. To explore the correlations with other gait and balance capacity measures in PD.
3. To examine the compliance and user-friendliness of the tracking devices in PD.

## 2. Materials and Methods

### 2.1. Participants

For this study, 28 PwPD and 18 HCs were prospectively recruited through a GDPR-compliant database of study volunteers between August 2018 and March 2021. Data of another fifteen HCs (total HC *n* = 33) were retrospectively obtained from a study in chronic obstructive pulmonary disease applying exactly the same methodological procedures [19]. Inclusion criterion for both groups were that they were aged 40 years or older. For PwPD, they also needed to have a diagnosis of idiopathic disease according to the UK Brain Bank criteria [20]. Exclusion criteria were: (I) other self-reported neurological diseases (than PD) or other conditions significantly affecting mobility; (II) having a body mass index (BMI) over 40 kg/m^2^; (III) using an assistive walking device; (IV) >1 fall per week in the past 6 months based on self-report; and (V) Mini-Mental State Examination (MMSE) score of <24/30. All participants provided written informed consent prior to participation in accordance with the Declaration of Helsinki and Ethical approval was obtained from the Ethical Committee Research UZ/KU Leuven (S60227).

### 2.2. Instruments

Three different commercial ATs were used in this study: the Fitbit Zip, Fitbit Alta, and Fitbit Inspire (Fitbit Inc., San Francisco, USA). The Fitbit Zip and Fitbit Alta were designed for wearing on the hip and wrist, respectively. The more recently developed Fitbit Inspire was designed for both locations and was brought into the study to replace defective Fitbit Zips and Fitbit Alta ATs, which were no longer available. All three commercial ATs entailed a triaxial accelerometer and proprietary algorithms to provide direct feedback to the wearer via the device display. The Fitbit Zip had a 3 V coin battery with an autonomy of 4 to 6 months. The Fitbit Alta and Fitbit Inspire had built-in batteries requiring charging every 5 to 7 days. The information displayed by the trackers was reduced to the minimum (step counts and clock) and the ATs’ built-in prompts and rewards were disabled. The research-grade monitor was the Dynaport Movemonitor (DAM—McRoberts BV, The Hague, the Netherlands), which was worn on the lower back with an elastic strap. The DAM contains a triaxial accelerometer, a triaxial magnetometer, a temperature sensor, and a barometer. It has a maximal measurement duration of 14 days without charging the battery at a sample frequency of 200 Hz. The DAM only records movement signals and does not run an onboard algorithm allowing direct feedback to the wearer. The DAM was previously validated in a laboratory setting for detecting step counts in PwPD compared to videotaped step counts (*n* = 32; ICC = 0.98; absolute percentage error 6.9 ± 3.0) [21,22]. Of note, however, is that short walks resulted in the highest absolute percentage error of step counts (3 m: 18.4 ± 21.0; 5 m: 9.6 ± 3.4). The DAM is currently used in an ongoing study to obtain regulatory endorsement for real-world digital mobility in PD and other chronic diseases [23].

### 2.3. Procedure

In this study, participants underwent a baseline assessment after which a 14-day activity monitoring period was started. During the baseline assessment, the following measures were collected in both groups: (1) demographics, (2) the Montreal Cognitive Assessment (MoCA), (3) the University of Alabama at Birmingham Life Space Assessment (LSA), (4) the 12 item Multiple Sclerosis Walking Scale (12-WS), and (5) a six-minute walk test (6 MinWT). PwPD, when ON-medication, also underwent: (1) the motor examination of the Movement Disorders Society Unified Parkinson Disease Rating Scale (MDS-UPDRS III), (2) the new-freezing of gait questionnaire (N-FOGQ), and (3) the MiniBESTest as a measure of balance capacity.

**Figure 1:**
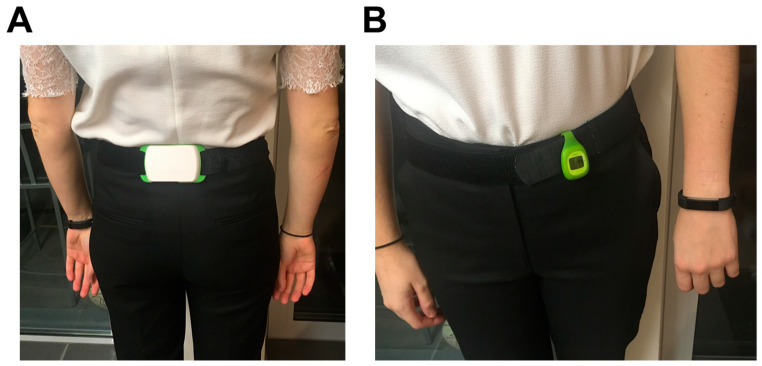
Placement of the DAM at the lower back (panel (**A**)) and the Hip-AT and Wrist-AT (panel (**B**)).

Next, participants were instructed to wear both an AT and the DAM for 14 days during wake time except for during bathing, showering, or swimming. No specific instructions were given to monitor their step counts regularly during use of the AT. Each participant wore a Fitbit Zip/Fitbit Inspire at the hip (Hip-AT) and a Fitbit Alta/Fitbit Inspire at the wrist (Wrist-AT). AT settings were adjusted according to the age, height, and weight of the participants and were worn on the same body side (see Figure 1). The HC participants wore the AT on their non-dominant side to reduce noise due to other arm movements. The DAM was positioned at the lower back and fastened by a strap; see Figure 1. PwPD wore the commercial AT on their least affected side (wrist or hip) as determined by the MDS-UPDRS-III. Participants received a visual demonstration on how to put on and recharge the AT. They also received a manual describing all the information for home use. Although batteries could last for multiple days, participants were instructed to recharge them each night to avoid step count discrepancies between the AT and DAM due to battery depletion. After the 14-day monitoring period, the devices were re-collected. A brief exit questionnaire (see Supplementary Materials), developed in our center [19], evaluated the user experience on a 5-point Likert scale including the following items: (1) the comfort of wearing, (2) recharging the AT, (3) how often they looked at the AT display, (4) for how long they would wear the AT in future daily routine, and (5) which AT they preferred. Finally, open questions were included to list the ATs’ positive and negative aspects.

Daily step counts were extracted from the online Fitbit platform after re-collecting the commercial devices. The DAM-data were uploaded and processed on the McRoberts cloud service, generating activity reports which included both the step counts and wearing time. All data were manually extracted from the respective platforms and entered into REDCap (www.project-redcap.org). Wearing time was only registered by the DAM. Only days with a wearing time of 8 h or more and only participants with at least 3 days of valid step count data from both the AT and the DAM were included for analysis [19].

### 2.4. Statistical Analysis

Normality was tested using Shapiro–Wilk tests and inspecting histograms and Q–Q plots. Parametric statistics were applied for all analysis. In case of abnormally distributed data, non-parametric statistics were also applied. If both parametric and non-parametric analyses showed similar results, parametric results are reported.

Criterion validity for daily step counts of the commercial AT was assessed through a 2 × 3 (group × device) ANOVA and absolute agreement intraclass correlation coefficients (ICC_2,1_) between each AT and the DAM per group. Bland–Altman plots were used to visually investigate the agreement between the AT and the DAM.

To evaluate whether the ATs were able to monitor day-to-day fluctuations, the delta between the step counts for each consecutive day was calculated. Next, for each subject, this delta was averaged and expressed as a percentage of the subject’s average step count over the 14-day period. A 2 × 3 ANOVA was used to test the differences between groups and ATs for this daily variance. In addition, the step counts for each participant and for each device were ranked separately from the most to the least active day. Next, the consistency of the ranking between the DAM and that of the AT was investigated using a Kendall correlation. Correlations were interpreted as: weak correlation r = 0.30–0.49; moderate correlation r = 0.50–0.69; strong correlation r = 0.70–0.89; and very strong correlation r ≥ 0.90 [24].

To explore the concurrent validity of AT-based step counts, Pearson correlations were used to examine the association with disease measures and gait and balance capacity outcomes in PwPD only. Differences in compliance between PwPD and HCs were calculated using an independent *t*-test on the average number of days of use and average wearing time. User experience and preferences were analyzed in PwPD only. Questionnaire data were analyzed descriptively as in the original publication [19]. Positive and negative statements were counted and added to the results only to help interpretation of the Likert-scale rated items.

All data were analyzed using SPSS version 28.0 (IBM, Armonk, NY, USA) and the significance level was set at *p* < 0.05 for all analyses. In case of significant interaction effects in the ANOVA analyses, Bonferroni corrected post hoc tests were applied. Exploratory correlation analyses were not corrected for multiple testing.

## 3. Results

Three HC participants were excluded because they had less than three valid days of step count. Demographics are presented in Table 1, showing that the groups were matched for age. As expected, PwPD had a significantly shorter 6 MinWT distance and higher subjectively reported walking difficulties on the 12-WS compared to the HC. Twelve PwPD self-reported having freezing of gait. Furthermore, the life space assessment tended to be smaller for PwPD than for HC (*p* = 0.06).

### 3.1. Criterion Validity

**Figure 2:**
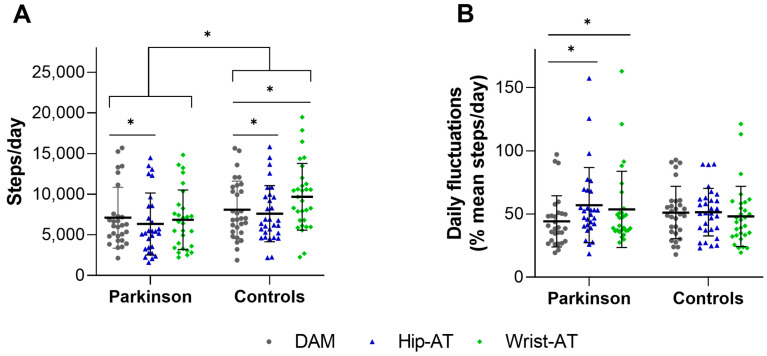
**Differences between DAM and both ATs in PwPD and HCs for steps/day (panel (A)) and daily fluctuations (panel (B)).** Each individual participant’s mean steps/day (panel (**A**)) and mean daily fluctuation (panel (**B**)) is represented with a point, for the DAM (grey dots), Hip-AT (blue triangles), and Wrist-AT (green diamonds). Thick horizontal lines mark the group’s mean and the whiskers show the SD. * Indicates significant differences. Hip-AT = Fitbit Zip or Fitbit Inspire; Wrist-AT = Fitbit Alta or Fitbit Inspire; DAM = Dynaport Movemonitor.

**Figure 3:**
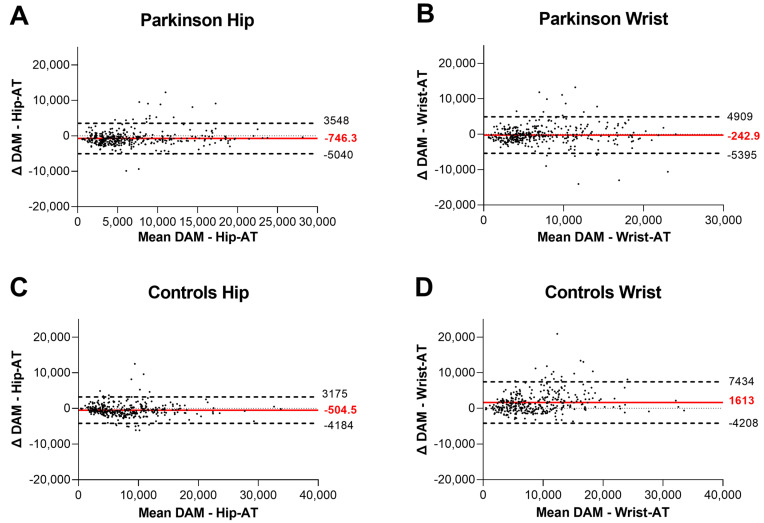
**Bland–Altman plots with mean and 95%CI for Hip-AT and Wrist-AT compared to the DAM.** Panels (**A**,**B**) show PwPD; panels (**C**,**D**) show the HC participants. Dots represent the daily steps for an individual day, irrespective of participant; small dotted line represents the zero (no difference); dashed lines represent the 95%CI with their accompanied values; full red line represents the bias in steps/day of the activity tracker compared to the DAM. Hip-AT = Fitbit Zip or Fitbit Inspire; Wrist-AT = Fitbit Alta or Fitbit Inspire; DAM = Dynaport Movemonitor.

Overall, there was a significant interaction effect (*p* < 0.001, see Table 2 and Figure 2A), with between-group post hoc tests revealing that both ATs measured fewer steps/day in PwPD compared to HCs, a discriminative ability which was similar to that of the DAM (*p* < 0.01). Within the DAM PwPD reached 87% of the HC values, within Hip-AT 83%, and within Wrist-AT 70% of HC values. Furthermore, the Hip-AT significantly underestimated the steps/day compared to the DAM, and this in both PwPD and HCs (ΔPwPD: −746 (−10%) steps/day; *p* < 0.001; Figure 3A, ΔHC: −505 (−6%) steps/day; *p* < 0.001; Figure 3C). In contrast, while the Wrist-AT significantly overestimated the steps/day in the HC (Δ 1613 (20%) steps/day; *p* < 0.001; Figure 3D), there was no significant difference with what the DAM found in PwPD (Δ −243 (−3%) steps/day; *p* = 0.29; Figure 3B). Despite these errors, overall daily step counts derived from the AT had good to excellent agreement with the step detections of the DAM (ICC_2,1_ > 0.83, see Table 2). These findings are supported by the Bland–Altman plots depicted in Figure 2. Interestingly, although the bias of the Hip-AT (−746 steps/day; Figure 3A) was larger than that of the Wrist-AT (−243 steps/day; Figure 3B), the 95% confidence intervals were smaller for the Hip-AT in PwPD, resulting in better ICC-values.

### 3.2. Detection of Daily Fluctuations

As presented in Table 3 and Figure 2B, a significant interaction effect was observed for the daily fluctuations (*p* = 0.005). Post-hoc analysis revealed that both the Hip-AT (*p* < 0.001) and the Wrist-AT (*p* = 0.03) significantly overestimated the daily fluctuations with 12.8% and 9.5%, respectively, in comparison with the DAM in PwPD only. No significant differences were observed between HCs and PwPD within each device (all *p* > 0.20).

**Figure 4:**
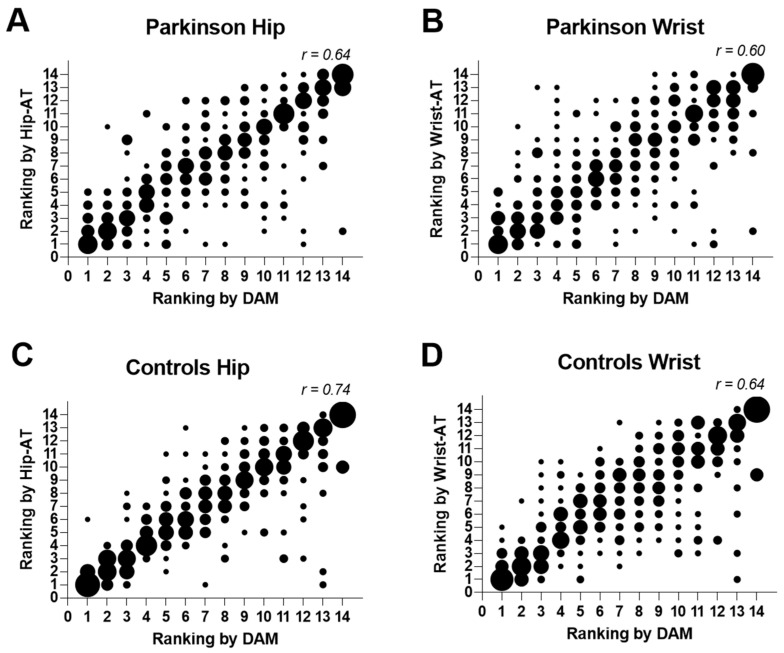
**Ranking of steps/day by DAM compared to ranking by activity trackers.** In PD, DAM compared to Hip-AT (panel (**A**)) and compared to Wrist-AT (panel (**B**)). In HC, DAM compared to Hip-AT (panel (**C**)) and compared to Wrist-AT (panel (**D**)). The larger the dot, the more subjects for the given combination of ranks. r = Kendall correlation for consistency of ranking. Hip-AT = Fitbit Zip or Fitbit Inspire; Wrist-AT = Fitbit Alta or Fitbit Inspire; DAM = Dynaport Movemonitor.

In line, the Kendall analysis indicated that the ranking of high- and low-step days was significantly associated between each commercial AT and the DAM, although less optimally in PwPD (Hip AT: r = 0.64; *p* < 0.001, Wrist-AT: r = 0.60; *p* < 0.001) compared to HCs (Hip-AT: r = 0.74; *p* < 0.001, Wrist-AT: r = 0.64; *p* < 0.001). In Figure 4, the larger dots indicate that more participants received a similar ranking from the ATs versus the DAM. PwPD had more scattered ranking in both Hip-AT (Figure 4A) and Wrist-AT (Figure 4B) in comparison with HCs (Figure 4C and Figure 4D, respectively). This worse scatter was more prominent in the Wrist-AT in comparison with the Hip-AT, although this ranking difference between ATs was similar in HC.

### 3.3. Concurrent Validity

Both the mean step count measure by Hip-AT and the Wrist-AT outcomes in PD correlated significantly with the 6 MinWT (both: R = 0.55; *p* < 0.01), which was similar for the DAM (R = 0.56; *p* < 0.01). Similarly, all three devices’ step counts correlated significantly with the Mini-BESTest (Hip-AT: R = 0.50; *p* < 0.01, Wrist-AT: R = 0.45; *p* = 0.02, DAM: R = 0.51; *p* < 0.01) and the LSA (Hip-AT: R = 0.50; *p* = 0.006, Wrist-AT: R = 0.43; *p* = 0.02, DAM: R = 0.39; *p* = 0.04). A weak correlation was seen for the 12-WS (Hip-AT: R = −0.32; *p* = 0.10, Wrist-AT: R = −0.33; *p* = 0.09, DAM: R = −0.34; *p* = 0.08) for all three devices. As for disease measures, both the Hip-AT and Wrist-AT step counts correlated weakly with MDS-UPDRS III (Hip-AT: R = −0.35; *p* = 0.07, Wrist-AT: R = −0.30; *p* = 0.12). Note that these correlations were not significant, in contrast to those from the DAM (R = −0.45; *p* = 0.02). The MoCA-scores were weakly correlated with the step counts of the Hip-AT (R = 0.30; *p* = 0.12) and DAM (R = 0.37; *p* = 0.05) but not correlated with the Wrist-AT step counts (R = 0.19; *p* = 0.34). Daily fluctuations calculated for all devices were not significantly associated with outcomes of physical capacity or disease severity.

### 3.4. User Experiences

Compliance with the AT was comparable between HCs and PwPD with an average daily wear time of 13.56 (1.70) hours in PwPD and 14.12 (1.61) hours in HCs (*p* = 0.20). However, the number of valid days was higher in PwPD (13.25 ± 0.93) in comparison to HCs (11.70 ± 2.65; *p* = 0.005). No differences between the number of valid days were determined between the retrospectively (11.71 ± 2.08) and prospectively (11.69 ± 2.99; *p* = 1.00) included HCs. Note that the three excluded HC participants for insufficient days of data were not included in this analysis.

Table 4 details the user experiences in PwPD only. Wearing the Wrist-AT was considered to be pleasant more often and the number of steps/day was more frequently checked on this device compared to the Hip-AT. In a similar vein, 21 (75%) of PwPD preferred the Wrist-AT, while only six (21%) preferred the Hip AT. Four of the 6 PwPD with a preference for the Hip-AT indicated that this was because of their fine motor difficulties in strapping on the Wrist-AT. The other two PwPD were surprised by the differences in the step counts between the Hip-AT and Wrist-AT, and had the impression that the Hip AT was more accurate. Only 11 (39%) PwPD were willing to use the Wrist-AT for a year at least. Thirteen PwPD were unwilling to use the Hip-AT again and for six PwPD the same applied for the Wrist-AT. Those unwilling to use the Wrist-AT were also unwilling to use the Hip-AT. Seven PwPD were willing to continue with the Wrist-AT, of which four were even willing to do this for a year at least. Overall, 22% of the PwPD disinclined further use of an AT in daily life.

## 4. Discussion

This study investigated the validity and user experience of commercial ATs for step count monitoring in PwPD. We contrasted the ATs’ ability to measure step counts to that of a research-grade device by comparing their performance between PwPD and HCs. Contrary to our hypothesis, step count measurement was worse in HCs compared to PwPD for the Wrist-AT, showing a consistent overestimation by 20% in HCs. Even though the Hip-AT significantly underestimated the number of steps, there was excellent agreement between the step counts of this Hip-AT with the research-grade monitor in PwPD, in contrast to the Wrist-AT. Otherwise, a largely similar pattern of good agreement between devices was found between and within groups. Furthermore, the between-group post-hoc analyses indicated that the ATs were able to discriminate PwPD from HCs as well as the DAM. These results are encouraging as they were derived from prolonged daily life walking for 14 days, constituting a unique feature of this study. Previous work also found valid results when comparing ATs with different ground truths, i.e., investigator in situ counts and video-based step detection [14,15,16,17]. However, these reports relied on shorter measurement periods and limited gait protocols.

Another important result from this study was that the ATs were able to measure daily fluctuations of step counts. Although daily fluctuations were overestimated by both ATs in comparison with the DAM in PwPD, the ATs could rank more from less physically active days similarly to the DAM. Taken together, this means that caution is warranted when interpreting fluctuations between consecutive days, whereas progression over a period of 14 days can be discerned reliably. This ability holds promise for future use of commercial ATs in a therapeutic context. Two recent studies have shown that activity tracking in conjunction with therapeutic advice delivered remotely was able to impact gait and balance capacity [25] and prevent the decline of step counts in one year in a more severely affected subgroup of PwPD [13]. However, as a considerable group of PwPD (43%) indicated that they were not inclined to use the devices for months or years as part of a clinical routine, adopting ATs in a therapeutic setting for PA stimulation may not be that straightforward in PwPD. In line with prior work [11], ATs may need to be integrated into rehabilitation programs by PD-specialized healthcare professionals in order to achieve optimal PA levels.

We found significant correlations between step counts and the 6 MinWT, a well-reported test of prolonged walking capacity, and the Mini-BESTest, representing balance capacity. Furthermore, higher step counts were modestly associated with higher LSA scores, a measure of self-reported mobility. These associations were robust as they were observed across all three devices. These results are contrary to the outcomes from other studies that daily life step counts would represent a different construct than gait capacity measures [26]. The discrepancy may be attributed to the type of ATs in the present study that allowed participants to make use of feedback displays. Indeed, 79% of the PwPD used this function at least once a day. This may have encouraged participants to ‘live-up’ to their capacity level. If so, it will not have influenced the validity outcomes as ATs and the DAM were always worn together. Speculatively, it also underscores the potential of the AT as a motivational tool for therapeutic purposes. However, since this study was limited to 14 days, it remains unknown how long the possibility of a ‘boosting’ effect would be maintained without therapeutic follow-up. The lack of significant associations between step counts as derived from the commercial ATs and measures of disease severity in the present study indicates the need to use more refined outcomes from research-grade ambulatory monitoring devices, such as the DAM, as possible biomarkers of disease severity and progression [23].

Preference for wrist-worn devices concurs with other studies investigating compliance with wearable sensors. Even though Silva de Lima et al. [27] did not compare a wrist-worn device with others, they found that in 805 participants with PD, compliance with a wrist-worn device reached 62–68% hours/participant/day. This rate only declined by 23–26% after 13 weeks. The reason for liking the wrist-worn device appeared to be due to the ease of checking the number of steps. However, two PwPD in our study found it more difficult to apply the Wrist-AT versus the Hip-AT. Interestingly, the Wrist-AT seemed to overestimate the step counts, particularly in HCs, compensating for the PD-related underestimation of step counts. This overestimation in HCs could be attributed to the fact that upper limb activities, such as folding laundry, were erroneously detected as steps [28]. We attempted to minimize this by instructing the HCs to wear the Wrist-AT on their non-dominant hand. As PwPD are more restricted in manual tasks [29], this drawback might not have impeded step estimations as much in PwPD. To minimize the effect of reduced arm swing on Wrist-AT’s step count detection, we instructed the PwPD to wear the Wrist-AT on their least affected side. However, since then, another study has shown that step counts from the more affected wrist may be more accurate [30]. Future studies need to examine why the affected arm enhances the accuracy of the detection. Possibly, this may be because the stationary arm is nearer the body’s center of mass [28] and thus closer to the spatial location of the DAM at the lower back. Yet, our lower accuracy for the Hip-AT compared to the DAM does not to support this notion.

Several limitations need to be considered when interpreting the present findings. In this study, we validated the commercial ATs against a research-grade DAM, which is considered a well-validated activity monitor available on the market [21,22]. Despite the fact that the DAM was previously validated for step measurement in PwPD, and is currently used in a large ongoing validation study including four different disease cohorts [23], most of its validation was conducted in a laboratory setting using straight-line walking. Only recently, the algorithms for the DAM’s step detection underwent further technical validation in a semi-structured and a daily life setting, improvements which were not yet available for implementation in the present study [31]. The commercial ATs under investigation in this study also did not allow access to the raw data of the internal sensor hardware, precluding passing through a technical validation framework described by Mazza et al. [31]. As a result, this study does not offer recommendations on how to improve the accuracy of the step count readings.

Although simultaneous use of the devices was a strength of this study, at the same time, participants were able to compare results, which may have influenced the subjective evaluation of the devices. In contrast to the daily wear-time of the DAM, the wear-time of the ATs could not be recorded objectively. Furthermore, valid days were based on the availability of data in all three devices, which could have been an underrepresentation of the actual compliance in wear time. PwPD were in the early to mid-stage of PD without cognitive impairment, having adequate activity levels, and this in a convenient sample size, which limits the generalizability of the findings to the wider population of PwPD. However, 43% of this cohort presented with freezing of gait and overall PwPD had lower step counts, suggesting that gait disorder was present in this group as expected. Finally, the differences between PwPD and HCs could be explained by the COVID-19 restrictions, possibly affecting the groups’ activity levels differently. All but three of the HCs were assessed prior to the COVID-19 pandemic, while this was only the case for seven of the 28 PwPD. Still, the within-subject comparison between the ATs and the research-grade monitor were not affected by the pandemic.

## 5. Conclusions

This study demonstrated that although commercial ATs lack some accuracy in registering daily step counts compared to a research-grade device, they have sufficient criterion validity for daily use in early- to mid-stage PwPD. We base this conclusion on the high agreement found between the ATs and the research-grade device for global step counts, as well as on their ability to differentiate high from low step counting days. The concurrent validity with other mobility outcomes also supports the use of ATs, as does the excellent compliance and adequate user-friendliness. About half of the PwPD indicated that they would consider continued use for a prolonged period. Therefore, we cautiously suggest that commercial ATs may be useful tools in therapeutic programs aimed to enhance daily PA levels. However, we also foresee that therapists’ input may be required to encourage more severely affected PwPD to apply ATs consistently to facilitate their long-term use.

## Figures and Tables

**Table 1:** Demographics.

	Parkinson Disease		Healthy Controls		Significance
	(*n* = 28)		(*n* = 30)		
Age (years)	66.	(8)	64	(8)	*p* = 0.39
Gender (M/F)	20/8		16/14		*p* = 0.18
BMI (kg/m^2^)	26.39	(3.14)	26.57	(3.28)	*p* = 0.83
6 MinWT (meters)	466	(100)	642	(69)	***p* < 0.001**
MoCA (0–30) *	27.21	(2.77)	27.19	(3.02)	*p* = 0.98
LSA (0–120) *	85.25	(21.68)	97.25	(14.69)	*p* = 0.06
12-WS (0–100) *	24.40	(20.16)	0.74	(1.43)	***p* < 0.001**
Disease duration (years)	9	(5)	/		
MDS-UPDRS III (0–132)	34.89	(9.60)	/		
LEDD (mg/day)	763.44	(409.33)	/		
MiniBESTest (0–28)	21.42	(3.57)	/		
N-FOGQ (0–27) ^#^	14.08	(6.56)	/		

M = male; F = female; BMI = Body mass index; 6 MinWT = 6 min walk test; MMSE = Mini mental state examination; MoCA = Montreal Cognitive Assessment; LSA = University of Alabama at Birmingham Life Space Assessment; 12-WS = 12 Item Walking Scale; MDS-UPDRS III = Movement Disorders Society Unified Parkinson disease rating scale motor examination; LEDD = Levodopa equivalent daily dosage; N-FOGQ = New freezing of gait questionnaire. * Healthy controls value only based on 16 prospectively assessed participants. ^#^ Based on 12 PwPD with N-FOGQ > 1. Significant *p*-values are indicated with bold. Values are presented as mean (standard deviation).

**Table 2:** Criterion validity.

	Parkinson Disease		Healthy Controls		Interaction Effect	Post-Hoc Group Effect
	(*n* = 28)		(*n* = 30)			
DAM	7187.38	(4933.67)	8198.52	(5272.05)	***p* < 0.001**	***p* = 0.008**
Hip-AT	6441.05	(5200.55)	7694.07	(5192.60)		***p* < 0.001**
Wrist-AT	6944.45	(5030.29)	9811.70	(5937.90)		***p* < 0.001**
Post hoc contrast *p*-value DAM—Hip-AT	***p* < 0.001**		***p* < 0.001**			
ICC_(2,1)_ DAM—Hip-AT	0.90	(0.86–0.92)	0.93	(0.91–0.95)		
Post hoc contrast *p*-value DAM—Wrist-AT	*p* < 0.29		***p* < 0.001**			
ICC_(2,1)_ DAM—Wrist-AT	0.86	(0.83–0.88)	0.83	(0.68–0.89)		

DAM = Dynaport Movemonitor; Hip-AT = Hip worn activity tracker; Wrist-AT = Wrist worn activity tracker; ICC = Intraclass correlation coefficient for steps/day. Significant *p*-values are indicated with bold. Values are presented as mean (standard deviation) steps/day or as ICC (95% confidence interval).

**Table 3:** Relative daily fluctuations.

	Parkinson Disease		Healthy Controls		Interaction Effect	Post Hoc Group Effect
	(*n* = 28)		(*n* = 30)			
DAM (%)	44.19	(19.88)	51.13	(20.40)	***p* = 0.005**	***p* = 0.20**
Hip-AT (%)	56.99	(29.19)	51.42	(18.55)		***p* = 0.39**
Wrist-AT (%)	53.64	(29.63)	48.09	(23.40)		***p* = 0.44**
Post hoc contrast *p*-value DAM—Hip-AT	***p* < 0.001**		***p* > 0.99**			
Post hoc contrast *p*-value DAM—Wrist-AT	***p* = 0.03**		***p* > 0.99**			

DAM = Dynaport Movemonitor; Hip-AT = Hip worn activity tracker; Wrist-AT = Wrist worn activity tracker; Significant *p*-values are indicated with bold. Values were calculated as mean (standard deviation) of the daily fluctuations relative to the average steps/day (%).

**Table 4:** User experiences in PwPD.

		Wrist-AT	Hip-AT
How pleasant was it to wear the tracker?	Pleasant	16 (57%)	7 (25%)
	Neutral	7 (25%)	18 (64%)
	Not pleasant	5 (18%)	3 (11%)
How frequently did you look at the step count values on the tracker?	Multiple times a day	16 (57%)	10 (36%)
	Once a day	6 (22%)	5 (18%)
	Once or twice a week	2 (7%)	5 (18%)
	Never	4 (14%)	8 (28%)
How long would you be willing to wear the tracker in the future as part of your clinical routine? *	A year or longer	11 (39%)	6 (22%)
	Months	5 (18%)	5 (18%)
	Weeks	4 (14%)	3 (11%)
	Days	1 (3.5%)	0 (0%)
	Never	6 (22%)	13 (46%)

* One PwPD did not respond to this question.

## Data Availability

The data presented in this study are available on request from the corresponding author. The data are not publicly available due to GDPR restrictions on pseudonymized participant information.

## Supplementary Materials

The following supporting information can be downloaded at: https://www.mdpi.com/article/10.3390/s23084156/s1. S1: Exit questionnaire.
