# Supplementary material for: Validation of Commercial Activity Trackers in Everyday Life of People with Parkinson’s Disease
Source: Sensors (Basel). 2023 Apr 21;23(8):4156. doi: 10.3390/s23084156 (PMC10144957; doi:10.3390/s23084156)
Supplement: Supplementary file 1 [file sensors-23-04156-s001.zip › sensors-2150294-supplementary.pdf]

‘Wearables’ for measuring physical activity

Participant experiences

For the past 2 weeks you have been wearing 2 wearables (1 at hip and 1 around the wrist), together with an activity monitor (at the lower back). With this short questionnaire we would like to check your experiences wearing these wearables. Your opinion is important for us to make activity programs even more patient friendly in the future. We ask you to take a moment to complete this questionnaire. Your input is greatly appreciated!

Thank you again for participating in this study and sharing your experience.

The following questions are about the **green wearable** worn at the **hip**.

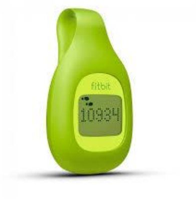

1) How comfortable did you find wearing this wearable?
    - Very pleasant
    - Pleasant
    - Neutral
    - No pleasant
    - No opinion
2) How often did you look at the steps on the wearable?
    - Multiple times a day
    - Once a day
    - Sometimes but not daily
    - Once or twice per week
    - Never
3) For what period would you like to continue using this wearable in the future?
    - More than one year
    - Several months
    - Several weaks
    - Several days
    - Never

4) What did you like about this wearable?

----

5) What did you dislike about this wearable?

The following questions are about the **black wearable** worn at the **wrist**.

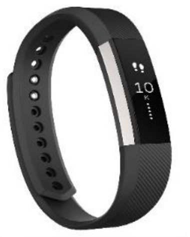

6) How comfortable did you find wearing this wearable?
    - Very pleasant
    - Pleasant
    - Neutral
    - No pleasant
    - No opinion
7) How often did you look at the steps on the wearable?
    - Multiple times a day
    - Once a day
    - Sometimes but not daily
    - Once or twice per week
    - Never
8) For what period would you like to continue using this wearable in the future?
    - More than one year
    - Several months
    - Several weaks
    - Several days
    - Never

9) What did you like about this wearable?

----

10) What did you dislike about this wearable?

----

11) How do you rate recharging the wearable?

    - Very easy
    - Easy
    - Not easy, but I managed it
    - Difficult
    - Very difficult

Which wearable would you prefer for future use (mark below)?

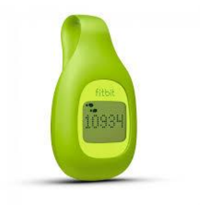

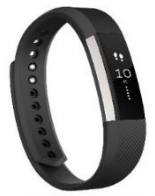

Neither one
